# Femtosecond to picosecond transient effects in WSe_**2**_ observed by pump-probe angle-resolved photoemission spectroscopy

**DOI:** 10.1038/s41598-017-16076-z

**Published:** 2017-11-22

**Authors:** Ro-Ya Liu, Yu Ogawa, Peng Chen, Kenichi Ozawa, Takeshi Suzuki, Masaru Okada, Takashi Someya, Yukiaki Ishida, Kozo Okazaki, Shik Shin, Tai-Chang Chiang, Iwao Matsuda

**Affiliations:** 1 Institute for Solid State Physics, the University of Tokyo, Kashiwa, Chiba, 277-8581 Japan; 2 Department of Physics, University of Illinois at Urbana-Champaign, 1110 West Green Street, Urbana, Illinois 61801-3080 USA; 3 Department of Chemistry, Tokyo Institute of Technology, Meguro-ku, Tokyo, 152-8551 Japan

## Abstract

Time-dependent responses of materials to an ultrashort optical pulse carry valuable information about the electronic and lattice dynamics; this research area has been widely studied on novel two-dimensional materials such as graphene, transition metal dichalcogenides (TMDs) and topological insulators (TIs). We report herein a time-resolved and angle-resolved photoemission spectroscopy (TRARPES) study of WSe_2_, a layered semiconductor of interest for valley electronics. The results for below-gap optical pumping reveal energy-gain and -loss Floquet replica valence bands that appear instantaneously in concert with the pump pulse. Energy shift, broadening, and complex intensity variation and oscillation at twice the phonon frequency for the valence bands are observed at time scales ranging from the femtosecond to the picosecond and beyond. The underlying physics is rich, including ponderomotive interaction, dressing of the electronic states, creation of coherent phonon pairs, and diffusion of charge carriers – effects operating at vastly different time domains.

## Introduction

Optical signal processing holds the key to developing ultrafast electronics. A fundamental question is how a material responds and relaxes as a function of time after a delta excitation by an optical pulse. Time-resolved and angle-resolved photoemission spectroscopy (TRARPES) is the most direct method of tracking the evolution of the electronic band structure, which also encodes the response of the lattice through electron-lattice or electron-phonon coupling. The research area has seen a rapid expansion centered on technologically promising materials such as graphene^1,2^, transition metal dichalcogenides (TMDs)^3^ and topological insulators^4,5^. Our study focuses on the short-time behavior of optical excitation of WSe_2_, which is a member of a vast TMD family, many of which exhibit novel properties that have galvanized the attention of the condensed matter physics community^6–8^.

**Figure 1:**
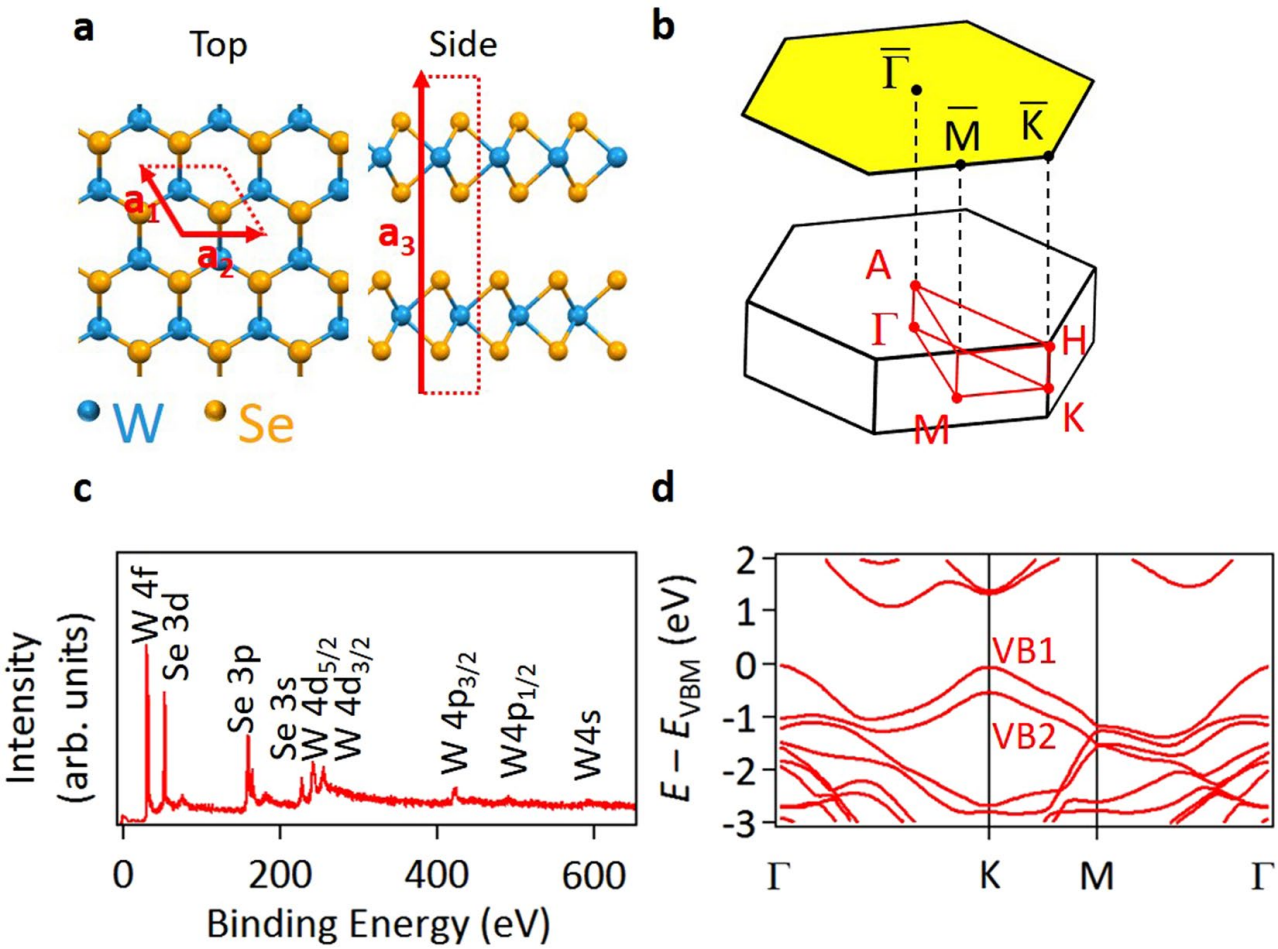
Crystal structure, Brillouin zone, and electronic structure of WSe_2_. (**a**) Top and side views of the atomic structure of 2H-WSe_2_. (**b**) First Brillouin zone and its planar projection. (**c**) X-ray photoemission spectroscopy data taken from a cleaved WSe_2_ crystal using 1200-eV photons. (**d**) Bulk band structure obtained by DFT calculations^11^.

The lattice structure of WSe_2_ (Fig. 1a) is made of Se-W-Se trilayers that are stacked via van der Waals forces in a 2H motif^9^; the periodicity along the layer stacking direction, *z*, is two trilayers. X-ray photoemission spectroscopy measurements of WSe_2_ (Fig. 1c) show sharp peaks derived from W and Se core levels with no contamination traces of C 1 *s* and O 1 *s* core levels. Referring Brillouin zones shown in Fig. 1b, the material is an indirect semiconductor with a gap of 1.22 eV between the valence band maximum at Γ and the conduction band minimum at the bottom of a “valley” between Γ and K (Fig. 1d)^10^. Its minimum direct gap, 1.64 eV, is located at the K point, where the two top valence bands (VBs) VB1 and VB2, separated by a spin-orbit splitting of 0.5 eV^11^, each exhibit a maximum. Direct optical absorption at the K point using polarized light can lead to selective valley population with a nontrivial Berry phase^12^. In our experiment, the pump laser photon energy, 1.55 eV, is below the direct gap, and optical absorption by indirect excitation is weak.

## Results

**Figure 2:**
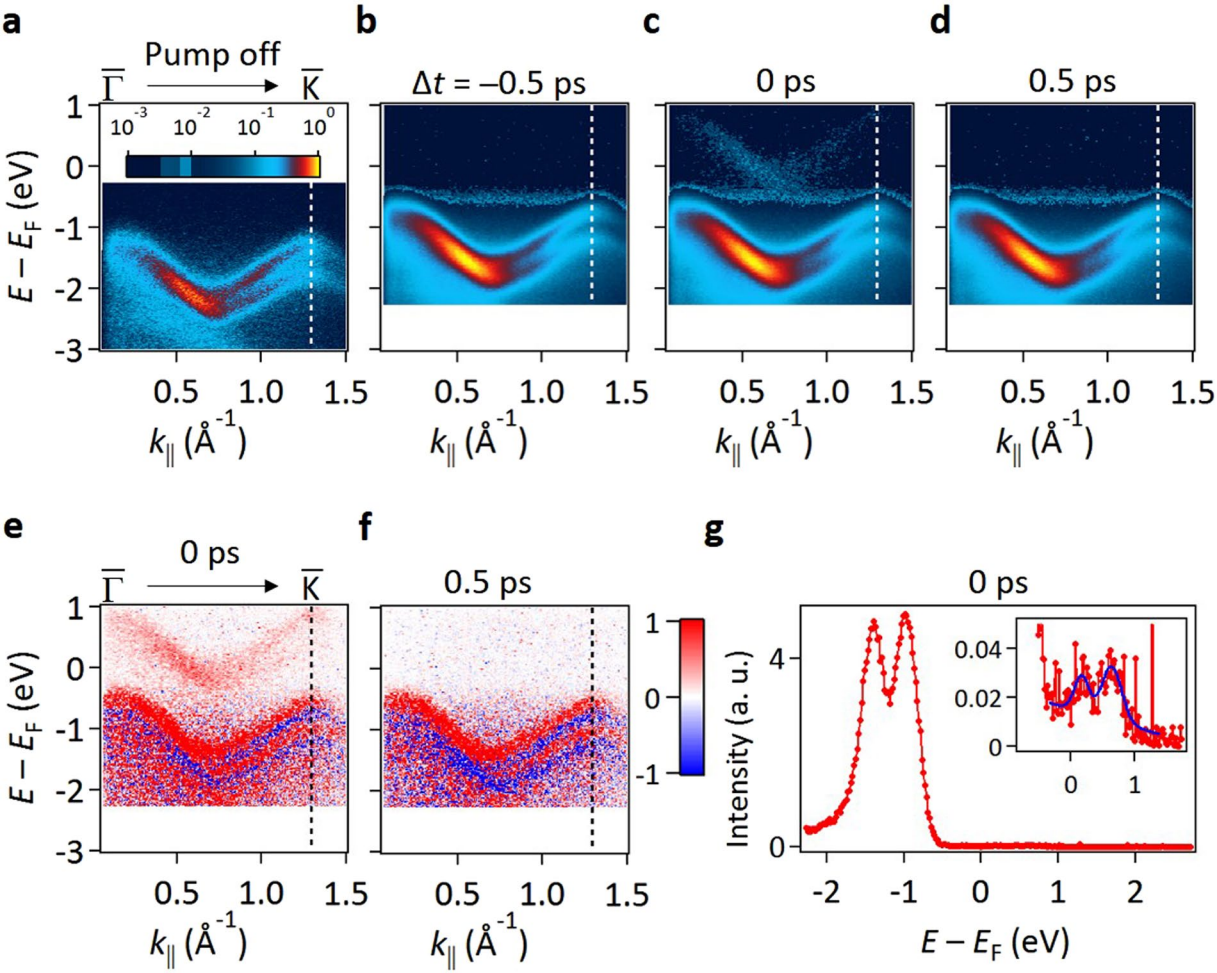
TRARPES maps of WSe_2_ along $\bar{{\rm{\Gamma }}}$ −$\bar{{\rm{K}}}$. (**a**) ARPES map without pump. (**b–d**) ARPES maps with pump on for pump-probe delay time Δ*t* = −0.5, 0 and 0.5 ps, respectively. (**e**) Difference map obtained by subtracting the Δ*t* =  −0.5 ps reference map from the Δ*t* = 0 map. (**f**) Difference map obtained by subtracting the Δ*t* = −0.5 ps reference map from the Δ*t* = 0.5 ps map. (**g**) EDC at $\bar{{\rm{K}}}$ for Δ*t* = 0. The inset highlights the *n* = +1 replica bands, where the blue curve is a fit.

Using a probe beam (84-fs pulses, 28-eV photons, 1-kHz rate), a spectrum obtained without application of the pump pulses (Fig. 2a) shows VB1 and VB2. The Fermi level, *E*_F_, locates close to the conduction band minimum, implying an n-type band alignment at the sample surface. With the pump pulses, the spectra at time delays of –0.5, 0, and 0.5 ps (Fig. 2b–d) reveal an approximately +0.5 eV shift of the bands toward *E*_F_. Further investigation confirms that the shift is independent of the pump-probe delay times over a wide range. The shift can be attributed to a surface photovoltage effect^13^; charge carriers created by pumping diffuse to flatten out the band bending near the surface, giving rise to a built-in voltage that shifts the bands. Since the carrier relaxation time is much longer than 1 ms, the surface photovoltage reaches a steady state in the experiment.

Additional spectral features are evident when the ARPES maps at delay times Δ*t* = 0 and 0.5 ps are subtracted by the ARPES map at Δ*t* = −0.5 ps. The difference maps should highlight the effects of pumping at the very short time scale. The results, with red and blue colors indicating positive and negative differences, show replicas of VB1 and VB2 (red bands) at 1.55 eV above the original bands for Δ*t* = 0 (Fig. 2e) but not for Δ*t* = 0.5 ps (Fig. 2f). The replica bands can also be seen in the original data (Fig. 2c). Additional time-resolved data indicate that the replica bands are instantaneously present during the pump pulse duration only. Similar replica bands have been reported in prior studies of Bi_2_Se_3_, where the replication of the topological surface states is attributed to a Floquet-Bloch process^4^. Our present study of WSe_2_ shows that similar effects can be observed for bulk Bloch states; surface concentration of the electronic wave function or nontrivial topological order is not a prerequisite for observing this phenomenon. Note that there is a very weak flat feature near the valence band maximum (about −0.5 eV) in Fig. 2b–d. Its intensity at the ~1% level become accentuated by the particular logarithmic color scale chosen for the presentation in order to bring out the very weak Floquet features. This feature likely arises from momentum non-conserving processes in ARPES that might include phonon and defect scattering.

## Discussion

In gas phase systems, replica states, generally called dressed or quasi-harmonic states, have been known since the 1970s^14^. The physics is that a strong pump field with photon energy $\hslash \omega$ can couple to an isolated electronic state at energy *E*, forming a ladder of states at energies $E+\frac{{e}^{2}{A}^{2}}{4m}+n\hslash \omega$, where *n* is an integer, and *A* is the vector potential of the pump field. This optical coupling occurs instantaneously during the pump pulse duration only. In our case, the red band seen in Fig. 2e corresponds to *n* = +1. The term $\frac{{e}^{2}{A}^{2}}{4m}$ arises from a ponderomotive interaction, and it leads to an energy shift of about 2–3 meV in our case. Figure 2g shows the measured energy distribution curve (EDC) taken at $\bar{{\rm{K}}}$. The intensity of the *n* = +1 replica (inset in Fig. 2g) is about 1% of the main line, in agreement with a theoretical estimate (see supplementary information). The energy separation of the *n* = +1 band from the main band agrees well with the pump photon energy of 1.55 eV. Higher replica bands including the next one at *n* = +2, predicted to have an intensity less than 10^−6^ of the main line, are too weak to be seen in our experiment. Dressing should also lead to an *n* = −1 replica of the same intensity as the *n* = +1 replica. It is indeed present in the data, but it is largely masked by photoemission from other overlapping valance bands and by secondary background from the main line (see supplementary information). Note that dressing of the states can also occur for the photoelectrons (Volkov process or laser-assisted photoemission^15^), leading to essentially the same spectral shapes. It can affect the replica band intensity determination, but its effect is generally not large and can be separated out or suppressed in the experiment (see supplementary information).

**Figure 3:**
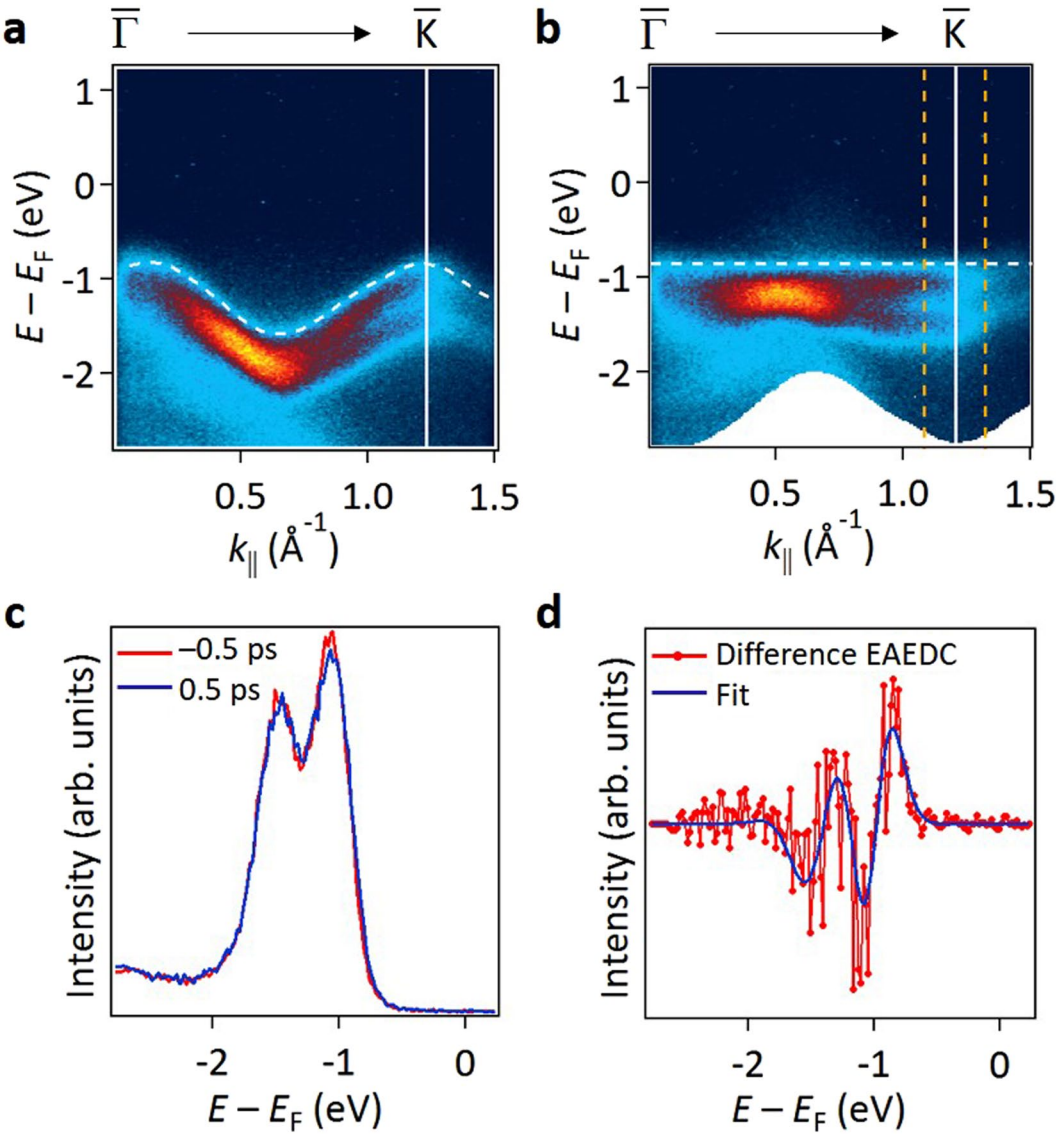
TRARPES data processing to highlight time-dependent evolution. (**a**) ARPES map for Δ*t* = −0.5 ps. The white dashed curve shows the energy dispersion of band VB1, arbitrarily shifted, as a reference. (**b**) ARPES map for Δ*t* = −0.5 ps shifted by the VB1 dispersion; the VB1 dispersion after the shift becomes a horizontal line. (**c**) EAEDCs for Δ*t* = −0.5 and 0.5 ps obtained by integrating the data over the region in *k* space marked by the two orange dashed vertical lines in **b**. (**d**) Difference EAEDC obtained by subtracting the Δ*t* = −0.5 ps EAEDC from the Δ*t* = +0.5 ps EAEDC in (**c**).

**Figure 4:**
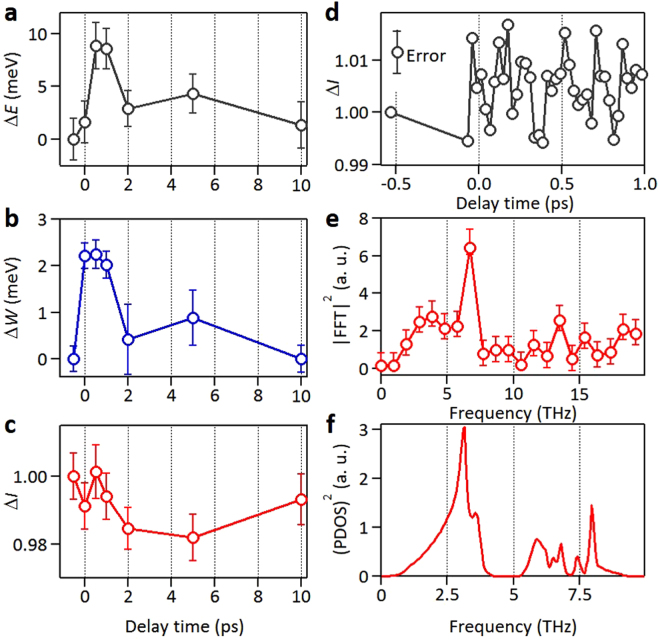
Energy shift, broadening, and intensity variation of the valence bands for various delay times. (**a**) Energy shift (Δ*E*), (**b**) broadening (Δ*W*), (**c**) normalized intensity variation (Δ*I*) of the VBs near $\bar{{\rm{K}}}$ deduced from fitting of EAEDCs for various delay times. (**d**) Another set of data of Δ*I* taken with a much finer time resolution. (**e**) Square of the fast Fourier transform (FFT) of Δ*I* in (**d**) as a function of frequency. (**f**) Square of the phonon density of states (PDOS)^21^ as a function of frequency as a measure of the joint density of states of coherent phonon pairs. The frequency scale here is one half of that in **e** to facilitate a comparison of peak positions in (**e**) and (**f**) that should be related by a factor two difference because of phonon pairing.

Bands VB1 and VB2 at Δ*t* = 0 and 0.5 ps, after subtraction of the data at Δ*t* = −0.5 ps, show adjoining red and blue regions (Fig. 2e,f); this is evidence for a positive energy shift (toward *E*_F_) and possibly other changes in the VB spectral shapes induced by the pumping. The evolution has a complex time dependence after the pump, unlike the instantaneous nature of the Floquet replica bands. To extract the time dependence with improved statistics, we integrate the ARPES data over a range in *k* space. To compensate for the band dispersion for the integration, the dispersion of each spectrum (Fig. 3a for Δ*t* = −0.5 ps) is “flattened” as a function of the wave vector by shifting the energy reference by the VB1 band dispersion. The resulting map (Fig. 3b) and the maps for other delay times are integrated over a range of wave vector near $\bar{{\rm{K}}}$ as indicated by the two vertical dashed lines in Fig. 3b, where VB1 and VB2 are sharp and well separated, to yield energy-aligned EDCs (EAEDCs); two such EAEDCs for Δ*t* = −0.5 and +0.5 ps are shown in Fig. 3c. They appear similar, but their difference (Fig. 3d) highlights changes by the pumping. The subtle changes can be well modeled by an energy shift (Δ*E*), an energy width broadening (Δ*W*) and a change in normalized intensity (Δ*I*) of the VBs (see supplementary information); the blue curve in Fig. 3d is a fit. The same analysis has been repeated for different delay times, and the extracted energy shift, broadening, and intensity variation are shown in Fig. 4a–c.

The energy shift of about +2 meV at time Δ*t* = 0 (Fig. 4a) can be attributed to the instantaneous ponderomotive interaction, and it agrees with our theoretical estimate. With a red detuning of the pump relative to the direct gap, a second-order optical Stark effect is expected to yield a negative instantaneous shift of the VBs^16^, but the magnitude is negligible under our experimental conditions. At later delay times, the energy shift rises to about 8 meV at Δ*t* = 0.5 and 1 ps before falling back down (Fig. 4a). The time scale corresponds well to coherent phonon excitations. While the pump pulse does not couple efficiently to single phonons except for those at the zone centers because of momentum conservation, it can readily create phonon pairs throughout the Brillouin zone^17^. At Δ*t* = 0, a broad-band coherent phonon-pair state (or a squeezed phonon state) is thus created. The atoms begin to move away from their equilibrium positions, resulting in a modulated crystal potential, which in turn leads to a shift in VB energies. The upswing of the VB energy shift after Δ*t* = 0 as seen in Fig. 4a can be attributed to this effect. The coherent phonons then propagate away from the excitation volume. Within ~10 ps, the phonon system should largely return to its quasi-static distribution, and the VB energy shift should diminish, as seen in the experiment.

In addition to the energy shift, the VBs exhibit a broadening (Fig. 4b), which attains a maximum value of about 2 meV at Δ*t* = 0−0.5 ps and decays at larger delay times. The broadening can be attributed to electron-phonon scattering. It is a maximum at and near Δ*t* = 0 ps due to the highest phonon population right after the pump. By contrast, the energy shift is zero at Δ*t* = 0 (other than the instantaneous ponderomotive interaction) because the atoms need time to move away from their initial equilibrium positions. The same adiabatic nature of atomic movements explains why the VB intensity is ~100% at Δ*t* = 0 (Fig. 4c). Once the atoms move away from their equilibrium positions, the broad-band squeezed coherent phonon state begins to unravel. A Debye-Waller-like effect sets in, which attenuates the intensity of the VB states (~5 ps in Fig. 4c). The intensity should recover smoothly once the phonon distribution returns to its quasi-static distribution (~10 ps in Fig. 4c). An interesting contrasting case is provided by a recent optical transmission study of coherent phonon oscillations in ultrathin films^18^, where ringing persists over a much longer time scale due to spatial confinement of the phonons.

To extract information about the coherent phonons, a much finer time resolution is needed for the first ~1 ps before decoherence occurs. Another set of data (Fig. 4d) for the VB intensity variation obtained by direct intensity integration over the region of interest near $\bar{{\rm{K}}}$ shows nontrivial variations at high frequencies. The square of the fast Fourier transform (FFT) of the intensity variation shows a strong peak at ~6.7 THz (Fig. 4e), which agrees well with twice the frequency of the main peak at 3.2 THz in the square of the phonon density of states (PDOS) (Fig. 4f). This factor of two correspondence is expected because optical excitation creates coherent phonon pairs with opposite momenta throughout the Brillouin zone. The square of the PDOS is just the 2-phonon joint density of states. The lattice is expected to oscillate at twice the phonon frequency, and the ARPES intensity should oscillate correspondingly.

The results from our TRARPES study reveal a rich dynamic behavior of WSe_2_. A number of fundamental processes are at play. At the shortest time scale, the system responds essentially instantaneously to the pump pulse via electronic state dressing by the Floquet process and via a ponderomotive interaction. Such dressing for bulk states (other than surface states), predicted to occur under optimal experimental conditions^19^, is seen here for the first time. Within the first several ps after the pump, the system responds in a complex manner to coherent phonon-pair creation and decay. At times up to milliseconds and beyond, diffusing charge carriers give rise to a surface photovoltage. The comprehensive characterization from this study of the time-dependent behavior over multiple time scales in a prototypical TMD provides a firm foundation for advancing optoelectronic technology and ultrafast electronics based on these and related materials.

## Methods

TRAPES measurements were performed using the system “4-Gouki” at the Institute of Solid State Physics of the University of Tokyo^20^. Pump pulses were produced using a 1-kHz Ti:Sapphire laser amplifier system with an output wavelength of 800 nm and a pulse width of 32 fs^20^. The second-harmonic output of the same laser was focused into an argon gas cell to yield the 9-th harmonic at 28 eV as the probe beam. The time resolution was about 84 fs as determined from the temporal response of a graphite reference sample. The size of the pump beam spot was 670 *μ*m. A WSe_2_ crystal purchased from HQ Graphene Co. was cleaved under ultrahigh vacuum to expose a fresh surface for the TRARPES measurements. All data were taken with the sample maintained at 40 K achieved by feedback-controlled liquid helium cooling. Initial sample characterization was performed at the X-ray Laboratory, the Institute for Solid State Physics, the University of Tokyo. X-ray photoelectron spectroscopy measurements were carried out using synchrotron radiation at beamline 13B of the Photon Factory, High Energy Accelerator Research Organization (KEK).

## Electronic supplementary material

Supplementary Info
