# Supplementary material for: Femtosecond to picosecond transient effects in WSe2 observed by pump-probe angle-resolved photoemission spectroscopy
Source: Sci Rep. 2017 Nov 22;7:15981. doi: 10.1038/s41598-017-16076-z (PMC5700159; doi:10.1038/s41598-017-16076-z)

Supplementary Information

Femtosecond to picosecond transient effects in WSe_2 observed by pump-probe angle-resolved photoemission spectroscopy

Ro-Ya Liu[1], Yu Ogawa[1], Peng Chen[2], Kenichi Ozawa[3], Takeshi Suzuki[1], Masaru Okada[1], Takashi Someya[1], Yukiaki Ishida[1], Kozo Okazaki[1], Shik Shin[1], Tai-Chang Chiang[2,1,*], and Iwao Matsuda[1,*]

1. Intensities of the Floquet-Bloch and Volkov replica bands

A straightforward calculation shows that the intensities for the $n = \pm 1$ Floquet-Bloch replicas due to dressing of the initial states are given by

$$I_{\pm 1} = I_0 \times J_{\pm 1}^2 \left(\frac{e}{2m\hbar\omega} \langle \boldsymbol{A} \cdot \boldsymbol{P} + \boldsymbol{P} \cdot \boldsymbol{A} \rangle \right) \times J_0^2 \left(\frac{e^2 |\boldsymbol{A}|^2}{8m\hbar\omega} \right) \qquad \text{(S1)}$$

where I_0 is the intensity of the original band, $<\ldots>$ denotes the matrix element in terms of the valence band states (VB1 and VB2) that can be computed readily using the known band structure and the effective mass approximation, J_0 and $J_{\pm 1}$ are Bessel functions of order 0 and ±1, respectively, m is the electron mass, ω is the angular frequency of the pump field and $\boldsymbol{A}$ is the vector potential of the pump field. The two replicas have the same intensity. The last factor $J_0^2 \left(\frac{e^2 |\boldsymbol{A}|^2}{8m\hbar\omega} \right)$ is very close to unity under our experimental conditions.

Dressing can also happen for the photoelectrons; this mechanism leads to replicas as well via the so-called Volkov or laser-assisted photoemission process. The same formula applies but can be simplified to:

[1]Institute for Solid State Physics, the University of Tokyo, Kashiwa, Chiba 277-8581, Japan, [2]Department of Physics, University of Illinois at Urbana-Champaign, 1110 West Green Street, Urbana, Illinois 61801-3080, USA, [3]Department of Chemistry, Tokyo Institute of Technology, Meguro-ku, Tokyo 152-8551, Japan. *e-mail: tcchiang@illinois.edu; imatsuda@issp.u-tokyo.ac.jp.

$$I_{\pm1} = I_0 \times J_{\pm1}^2\left(\frac{e\boldsymbol{A}\cdot\boldsymbol{v}}{\hbar\omega}\right) \times J_0\left(\frac{e^2|\boldsymbol{A}|^2}{8m\hbar\omega}\right) \tag{S2}$$

where $\boldsymbol{v}$ is the photoelectron velocity in vacuum. The resulting replica bands have the same dispersion relations as those arising from the Floquet-Bloch process. The Volkov process can be suppressed in our experiment by choosing a polarization configuration for which $\boldsymbol{A}\cdot\boldsymbol{v} = 0$. Under this condition and with $|\boldsymbol{A}| = 2.67\times10^{-7}$ Vs/m, corresponding to a pump fluence of 1.68 mJ/cm^2, we obtain from Eq. (S1) that

$$\frac{I_{\pm1}}{I_0} = 1.07\times10^{-2}$$

This is very close to our experimental value of $I_{+1}/I_0 = 9.1\times10^{-3}$, where I_{+1} (I_0) is approximated by the ARPES intensity integrated over k and over energy ranging from +0.05 to +1.55 eV (0 to –1.5 eV) relative to the valence band maximum.

2. Evidence for the n = –1 replica

Fig. S1a shows the theoretical band structure of WSe_2 (blue curves) and the expected +1 and –1 replica bands (red curves) arising from the two topmost valence bands. Fig. S1b shows the difference ARPES map obtained by subtracting the map at Δt = **–**0.5 ps from the map at $\Delta t = 0$. The red curves in Fig. S1b indicate the expected replica band positions based on the experimental dispersion relations of VB1 and VB2. For clarity, Fig. S1c shows the same difference map but without the overlapping red curves. The +1 replica bands are very clear, but the –1 replica bands are not as clear as the +1 replica bands because of noise arising from other overlapping bands and secondary background emission.

As a further test of the –1 replica, we show in Fig. S2a the difference ARPES map at Δt = 0 "flattened" by shifting the energies at each k points by the dispersion of band VB1 as was done for Fig. 3b in the main text. The +1 band of VB1 then appears horizontal. The map is then integrated over k to improve the signal to noise ratio, yielding the EAEDCs shown in Fig. S2b for different delay times. The +1 replica bands appear as two peaks in the difference EDCs for Δt = 0 and 67 fs, but the intensities for the latter case are reduced because the pump and probe only partially overlap in time. The difference EDC for Δt = 500 fs does not show the +1 bands, as expected. The –1 replica bands are evident and behave similarly as a function of delay time, although the peaks in the difference EDCs are distorted because of uncertainties arising from signals from the overlapping bands.

3. Extracting the energy shift, broadening, and intensity change of TRARPES spectra

The results shown in Fig. 4a-c are extracted from the data as follows. The EAEDC at Δt = –0.5 ps, $S_{-0.5}(E)$, is the reference. Each EAEDC at a given delay time $S_{\Delta t}(E)$ can be well described by an energy shift, a broadening, and a change in the normalized intensity relative to the reference. The difference EAEDC, $\Delta S(E) = S_{\Delta t}(E) - S_{-0.5}(E)$, is fitted to the following function:

$$(1 + \Delta I) \int S_{-0.5}(E + \Delta E - x) \times \mathrm{G}(x, \Delta W)\, dx - S_{-0.5}(E)$$

where G is a normalized Gaussian, and ΔE, ΔW, and ΔI are the energy shift, broadening, and normalized intensity change relative to the reference. The results of the fitting are shown in Fig. 4a-c.

As an illustration of the process, we show in Fig. S3a a reference EDC represented by a model function (blue curve) comprising of two Lorentzians to represent the two peaks arising from VB1 and VB2. A sample curve after convolution as described above (red curve), together with the difference (Fig. S3b) are also shown. This difference curve resembles the fitting curve in Fig. 3d.

4. Raw data and data reduction for TRARPES

Figure S4 shows representative raw data and data reduction for the intensity variations of TRARPES. During the experiment, ARPES maps were recorded as a function of delay time. The measurements were repeated over many cycles, and the results were summed to improve the signal to noise ratio. Figure S4a presents an ARPES map at Δt = –0.5 ps, while Fig. S4b is a reproduction of Fig. 4d in the main manuscript, included here for easy reference. Delay times Δt = –66, 66, 360, and 864 fs correspond to local minima in Fig. S4b, while delay times Δt = –40, 172, and 518 fs correspond to local maxima. At these representative delay times, the difference EDCs, integrated over the momentum range indicated in Fig. S4a, are shown in Fig. S4c. The change in intensity is up to about 5% of the original peak intensity of VB1.

Figure S1| Band structure and ARPES maps showing evidence for the +1 and –1 replica bands. a, Band structure (blue curves) of bulk WSe_2 along Γ to K. Red curves represent expected +1 and –1 replica bands separated from VB1 and VB2 by ± 1.55 eV. **b**, Difference ARPES map for Δt = 0. Red curves indicate the expected replica bands. **c,** Same difference ARPES map without the red curves presented here again for clarity. The fluence of the pump pulse for this experiment was 1.68 mJ/cm^2.

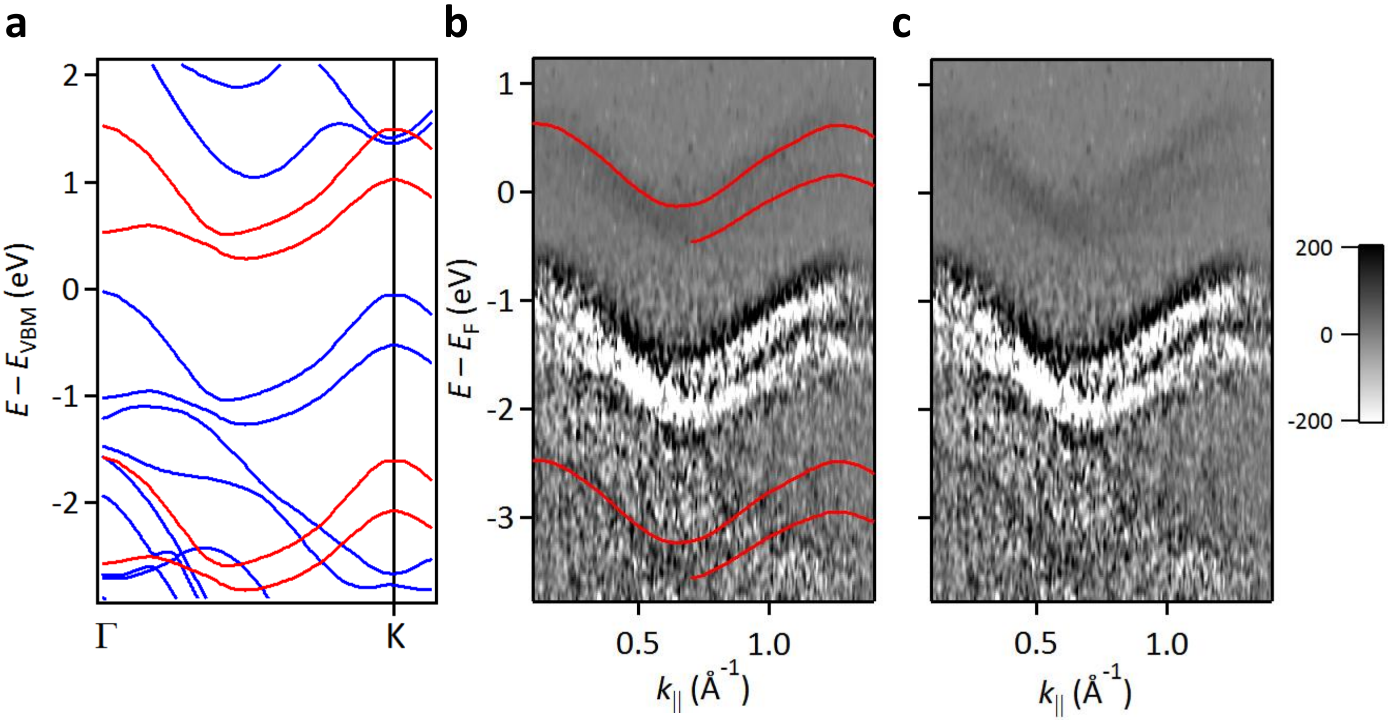

Figure S2 | Evidence for the *n* = –1 replica. a, Flattened difference ARPES map for Δt = 0. **b,** Red, green and blue curves indicate the difference EAEDCs for Δt = 0, 67, and 500 fs, respectively. These difference EAEDCs were obtained by integrating the difference ARPES maps over the entire range of *k* shown in **a**. The arrows indicate the expected band positions.

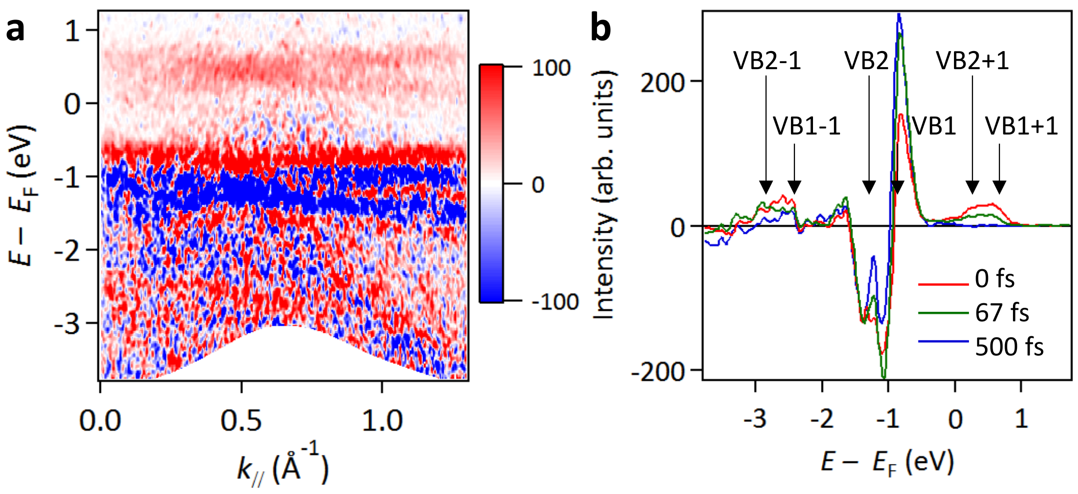

Figure S3 | Convolution fitting for extracting the energy shift, broadening, and intensity variation. a, Blue and red curves represent a reference curve (Δ*t* = –0.5 ps) and the same curve after the convolution described in the text that include an energy shift, broadening and intensity change. **b,** the difference between the two curves in **a**.

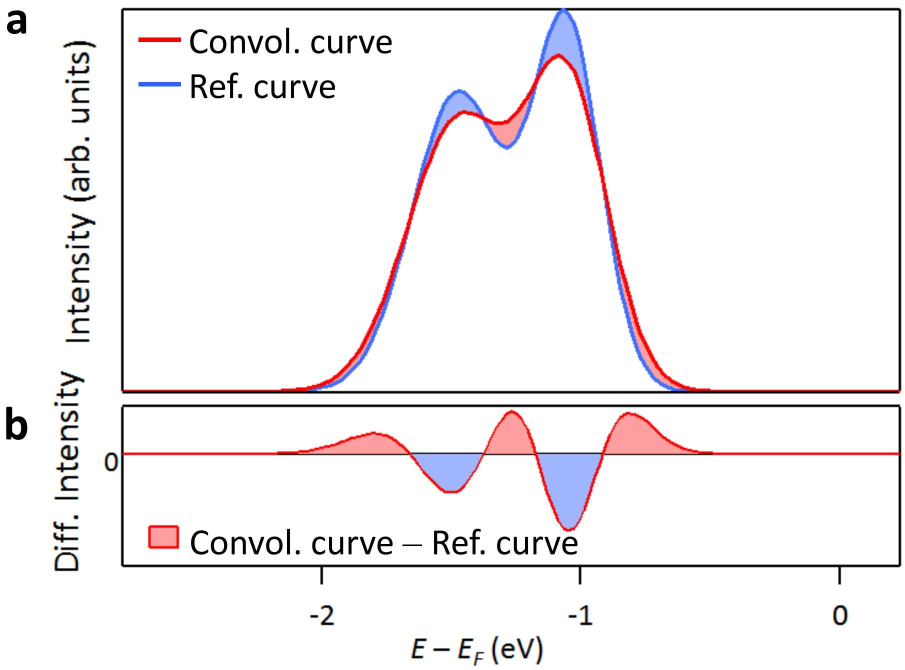

Figure S4 | Temporal intensity variations within the region of interest. a, ARPES map at Δt = –0.5 ps. **b,** reproduction of Fig. 4d from the main text included here for reference. **c**, difference EDC curves integrated over the range of k indicated by the two dashed vertical lines in **a** at different delay times with Δt = –0.5 ps as the reference. The chosen delay times Δt = –66, 66, 360, and 864 fs correspond to local minima in **b**, while delay times Δt = –40, 172, and 518 fs correspond to local maxima.

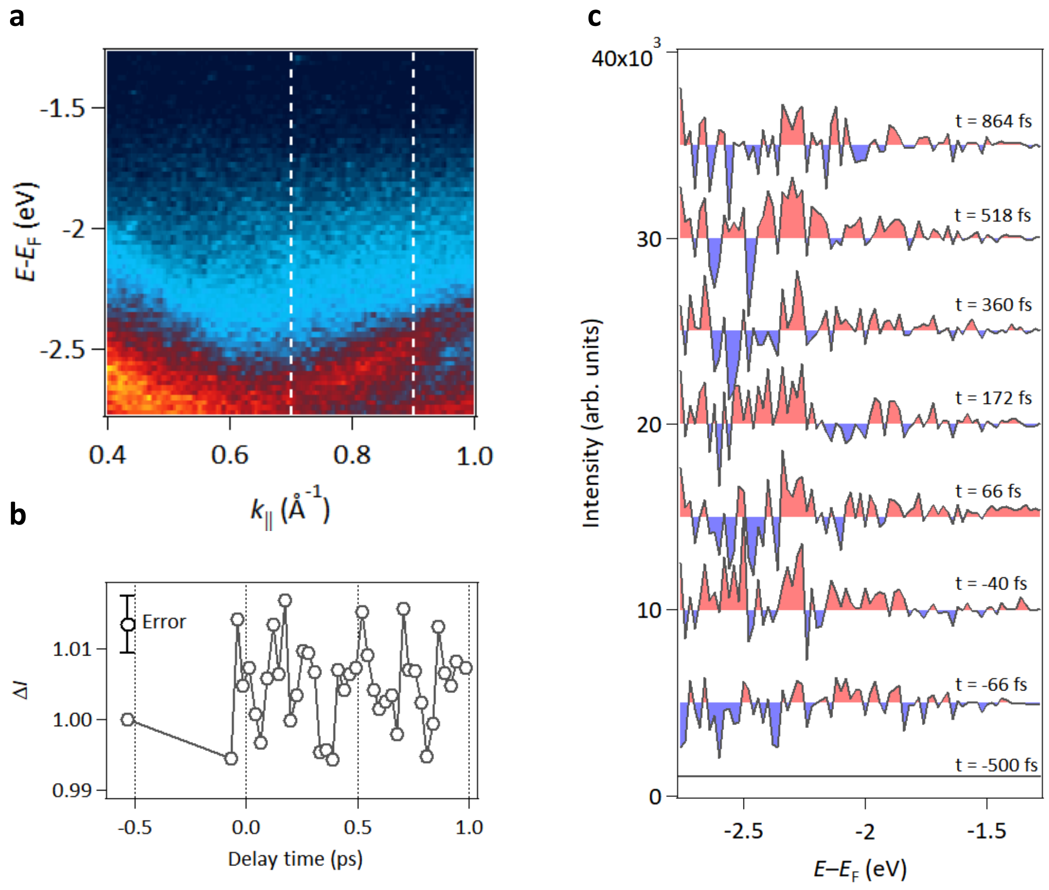

Supplement: Supplementary file 1 — Supplementary Info [file 41598_2017_16076_MOESM1_ESM.pdf]
